# BMI-Dependent Correlations of Sex Hormone Genes with Simple Endometrial Hyperplasia

**DOI:** 10.3390/life16060937

**Published:** 2026-06-02

**Authors:** Vladimir Churnosov, Maria Churnosova, Evgeny Reshetnikov, Inna Aristova, Kirill Tsoy, Inna Sorokina, Alexey Polonikov, Maria Solodilova, Mikhail Churnosov, Irina Ponomarenko

**Affiliations:** 1 Department of Medical Biological Disciplines, Belgorod State National Research University, 308015 Belgorod, Russia; 958561@bsuedu.ru (V.C.); churnosovamary@gmail.com (M.C.); reshetnikov@bsuedu.ru (E.R.); aristova@bsuedu.ru (I.A.); tsoy@bsuedu.ru (K.T.); sorokina@bsuedu.ru (I.S.); polonikov@rambler.ru (A.P.); solodilovama@kursksmu.net (M.S.); ponomarenko_i@bsuedu.ru (I.P.); 2 Department of Biology, Medical Genetics and Ecology, Kursk State Medical University, 305041 Kursk, Russia; 3 Research Institute for Genetic and Molecular Epidemiology, Kursk State Medical University, 305041 Kursk, Russia

**Keywords:** BMI, sex hormones, simple endometrial hyperplasia, SNP, association

## Abstract

The aim of the work was to identify the features of associations of single nucleotide polymorphisms (SNPs) that determine the level of sex hormones in the body with simple endometrial hyperplasia without atypia (EnH_s_) in women with different body mass index (BMI). Two groups of BMI-differing subjects (BMI < 25 [n = 766]: 196 EnH_s_, 570 control and BMI ≥ 25 [n = 727]: 324 EnH_s_, 403 control) with a total number of 1493 women were formed to conduct this study. Nine SNPs meaningful for the level of sex hormones (proven in genome-wide association studies [GWAS]) such as rs34670419 [G>T] *ZKSCAN5*, rs148982377 [T>C] *ZNF789*, rs11031002 [T>A] *FSHB*, rs11031005 [T>C] *FSHB*, rs112295236 [C>G] *SLC22A10*, rs117145500 [A>C] *CHD9*, rs727428 [C>T] *SHBG*, rs1641549 [C>T] *TP53*, rs117585797 [C>A] *ANO2* were considered by us. In general, in the studied sample (EnH_s_/control, n = 1493), an association of interactions of rs148982377 [T>C] *ZNF789* × BMI with EnH_s_ (OR = 1.11) was revealed. As a result of a comparative analysis, we found that rs148982377 [T>C] *ZNF789* was associated with EnH_s_ in women with a BMI ≥ 25 (OR = 1.77) and was not linked with the disease in women with a BMI < 25. Both common protective factors for EnH_s_ were also identified in women with BMI ≥ 25 and BMI < 25 (rs11031002 [T>A] *FSHB* (OR = 0.43 and OR = 0.46 respectively) and rs11031005 [T>C] *FSHB* (OR = 0.46 and OR = 0.58 respectively)). At the same time, in the BMI ≥ 25 cohort, the risk effect of the TT*rs11031002-rs11031005 haplotype (OR = 3.27) was more pronounced, and the contribution of interlocus interactions of sex hormone genes with EnH_s_ formation was more significant (7 SNPs in 12 models) than in the BMI < 25 group (OR = 2.31 and 4 SNPs in 3 models). In conclusion, the presented work is exploratory and demonstrates differences in the nature of the association with EnH_s_ in women with different BMI of the testosterone-determining polymorphism: SNP rs148982377 [T>C] *ZNF789* was associated with EnH_s_ in women with a BMI ≥ 25 and was not linked with the disease in women with a BMI < 25.

## 1. Introduction

Endometrial hyperplasia (EnH) is based on damage to the endometrium of the uterus due to a higher proliferation of glandular structures, leading to an imbalance in glandular-stromal ratios [1]. The prevalence of EnH in general among the female population (18–90 years old) reaches 132 per 100,000 women-years (simple EnH—58, complex—63, atypical—17 per 100,000 women-years) [2]. EnH is an age-dependent disease: disorder is occasionally recorded in women under 30 years of age (6 per 100,000 women-years), and its frequency reaches the highest level in the age categories of 50–54 years (simple (EnH_s_)—142, complex—212 per 100,000 women-years) and 60–64 years (atypical EnH—54 per 100,000 women-years old) [2]. The most frequent and pronounced symptom of EnH is abnormal uterine bleeding, which reduces the quality of life of women [3,4,5,6]. Among women with disease progression and the development of endometrial cancer within 1 year after the diagnosis of EnH, a 40% probability of malignant transformation for atypical hyperplasia and a 10% probability for GPE with the absence of atypia are shown [7]. The cumulative 20-year risk of disease progression is less than 5% in EnH without atypia and 28% in atypical EnH [8].

The role of obesity and overweight as a risk factor for EnH is beyond doubt [3,4,5]. In overweight women (BMI = 25–29.9 kg/m^2^), the risk of complex EnH is 3 times higher than in women with normal body weight, and in obese women (BMI > 30 kg/m^2^), these indicators are already 3.7 (for EnH without atypia) and 4.6 (for complex EnH) [9]. It is indicated that there are significantly higher risks of developing EnH in women with a BMI > 40 (13-fold for EnH with atypia and 23-fold for complex EnH) [9]. In patients with abnormal uterine bleeding with a BMI > 30, complex EnH or endometrial cancer are registered 4 times more often than in women with a lower BMI [10]. It is believed that with overweight/obesity, hyperestrogenism develops (due to an increase in the extra-gonadal synthesis of estrogens from androstenedione, a decrease in the level of sex hormone binding protein [SHBG], and the development of anovulation), leading to a more pronounced proliferation of endometrial cells [3,4,6,9,11]. Along with this, hormonal changes such as deficiency of progesterone effects, disturbances in the ratio of follicle-stimulating hormones (FSH) and luteinizing hormones (LH), androgen effects, etc., are important in EnH formation [3,4,5,12,13].

Genome-wide association studies (GWAS), especially those actively conducted in recent years, have established genetic determinants (SNPs) correlating with sex hormone concentrations and their metabolites (progesterone, estradiol, testosterone, SHBG, LH, FSH, dehydroepiandrosterone (DHEAS), etc.) in the organism [14,15,16,17,18,19,20,21,22,23,24]. On the one hand, a number of studies have shown numerous associations of GWAS-significant polymorphic variants (and strongly linked loci) that determine the level of sex hormones with the development of such hormone- and BMI-dependent signs/diseases of the female reproductive organs such as age at menarche/menopause, breast cancer, endometriosis, polycystic ovaries, uterine fibroids, ovarian cyst, etc. [25,26,27,28,29,30,31,32,33,34,35,36,37,38,39,40,41]; on the other hand, a significant modifying effect of BMI (obesity/overweight) on the SNP–disease correlations in various diseases has been demonstrated [42,43,44,45,46]. For EnH, despite the paramount importance of both sex hormones and obesity/overweight in the pathophysiology of this disease, no similar studies have been conducted to date.

It should be noted that the number of genetic studies of EnH is extremely small—only a few studies have been conducted aimed at finding associations of the disease with the SNPs of limited groups of candidate genes (menarche, apoptosis, estrogen receptors, growth factors, cytochromes, tumor necrosis factors, chemokines, interleukins, etc.) [47,48,49,50,51,52]; heritability indicators of EnH are not known, and GWAS have not been conducted. Thus, at the moment there is no single unambiguous understanding of the genetic determinants of EnH, and the few genetic data obtained on this issue are fragmentary, often contradict each other, and do not take into account the combined influence of such risk factors for EnH_s_ development as obesity and SNP candidate genes, which determines the relevance of this work.

## 2. Materials and Methods

### 2.1. Study Subjects

When planning this work, we calculated the number of samples (EnH_s_/control) in each of the studied subgroups of women (BMI < 25; BMI ≥ 25) necessary to obtain representative results (required power = 80%, α = 0.05, establishment of differences in the frequencies of polymorphic variants between “EnH_s_-control” in the framework of the additive model at the level of OR = 1.35–1.60). Calculations performed using the Quanto program (version 1.2.4) indicate that the total sample size in each of the considered subgroups (BMI < 25; BMI ≥ 25) should be at least 700 women.

Two groups of BMI-differing subjects (BMI < 25 [n = 766]: 196 EnH_s_, 570 control and BMI ≥ 25 [n = 727]: 324 EnH_s_, 403 control) with a total number of 1493 women were formed for this study. The sample for the study was formed in 2006–2013 by qualified gynecologists of the perinatal center at the Regional Clinical Hospital based on the following inclusion criteria [53,54,55,56,57]: (a) were born [lived] in Central Russia; (b) Russian nationality (self-identification was carried out); (c) were not relatives; (d) gave voluntary consent to participate in the study. The exclusion criteria were: (a) non-Russian nationality; (b) born [living] outside Central Russia; (b) having severe chronic pathology of vital organs, oncopathology, or serious autoimmune disorders; (c) refusal to participate in the study. The EnH_s_ group included patients with a disease diagnosis such as simple endometrial hyperplasia without atypia verified by morphological method (endometrial tissue obtained as a result of hysteroscopy or curettage was analyzed) [52,58]. The control group was formed during the same period of time (2006–2013) and included individuals without any (clinical, anamnestic, ultrasound) manifestations of female reproductive organ (pelvis) pathology [52]. The research design was reviewed in detail at the planning stage and approved by the specialists of the regional ethics committee.

It should be emphasized that only EnH_s_ was studied in this work. This is due to the fact that this is a single histological subtype of EnH, which makes it possible to form a homogeneous (by histological subtype) sample of patients and thereby reduce the risks of obtaining distorted estimates of associative relationships due to the heterogeneity of the sample. Also, the simultaneous inclusion of several samples of patients with different histological subtypes would significantly increase the likelihood of false positive results, which would require additional adjustments for multiple comparisons, which in turn would lead to false negative results.

### 2.2. SNPs Laboratory Examination

The work used DNA obtained by us in a previously performed study (2008–2013) of EnH_s_ candidate genes [52]. DNA stored in Kelvinators at t = −80 degrees in the Belgorod State University biobank. DNA samples were stored in Kelvinators at a low temperature (t = −80 °C) and had the required degree of purity (ratio 260 nm/280 nm, in the range of 1.7–2.0), which was monitored during the preparation of DNA samples for genetic testing. For genotyping from stock DNA samples (highly concentrated DNA stored in a biobank), a working collection of DNA samples in concentrations ranging from 10–20 ng/mL was prepared. Measurements of DNA purity and concentration during the preparation of the working collection were carried out on a NanoDrop™ 2000 device (Thermo Fisher Scientific, Waltham, MA, USA).

Nine polymorphic gene variants meaningful at the level of sex hormones (proven in GWAS, Table S1) such as rs34670419 [G>T] *ZKSCAN5*, rs148982377 [T>C] *ZNF789*, rs11031002 [T>A] *FSHB*, rs11031005 [T>C] *FSHB*, rs112295236 [C>G] *SLC22A10*, rs117145500 [A>C] *CHD9*, rs727428 [C>T] *SHBG*, rs1641549 [C>T] *TP53*, rs117585797 [C>A] *ANO2* were considered by us. The aforementioned SNPs have considerable functionality (HaploReg data [59], Table S2) and are correlated with the organism level of the following sex hormones: total and bioavailable testosterone, its metabolites [17,19,20,21,22,23], estradiol [16,19], DHEAS [15,16], cortisol/DHEAS ratio [15]), SHBG [14,16,20,24], free androgen index (FAI) [16,22], progesterone [16], FSH/FSHB [16,18], and LH [16]. Real-time PCR (TaqMan method) [60,61], implemented in a CFX96 device (Bio-Rad Laboratories, Hercules, CA, USA), was used to obtain experimental data on the EnH_s_/controls genotypes at the 9 loci under consideration. To confirm the quality of the laboratory data obtained, positive/negative control samples were used during each polymerase chain reaction (96-well plates were used), and blind re-genotyping of DNA samples randomly taken from the total EnHs/controls sample (about 5%) was also performed [62,63].

### 2.3. Statistical Analysis of Genetic Data

Previously, before carrying out the associative analysis, a comparison of the observed and expected values in accordance with HWE in the distribution of genotypes at all loci in all studied groups [BMI < 25; BMI ≥ 25 and EnH_s_; control] was performed [64]. In order to minimize errors associated with false-positive results (due to multiple testing), we introduced the necessary adjustments for multiple comparisons at each stage of the study.

At the initial stage of the study, we analyzed the associations of SNP × BMI interactions with EnH_s_ using the logistic regression method (association parameters [OR, 95%CI] [65,66,67] were calculated), implemented in the gPlink program (v. 1.07) [68], in a combined sample of patients (n = 520) and controls (n = 973). BMI was included in the analysis as a quantitative variable. The calculations were performed using the additive genetic model, which is the most commonly used in genetic research (including GWAS). To minimize false-positive results associated with multiple comparisons in the analysis of 9 SNPs, we used a permutation test and calculated the p_(perm)_ indicator [69]. The permutation test is effective for solving the problem of multiple comparisons when analyzing large arrays of GWAS data without reducing power [69]. The results were considered statistically significant at p_(perm)_ ≤ 0.05. The Bonferroni correction was not introduced here, since only one comparison pair (patients/controls) was analyzed within the framework of one genetic model (additive), adjusted for the number of analyzed SNPs (n = 9) due to permutation testing.

To identify the BMI-dependent peculiarity of “EnH_s_-SNP” connections, we conducted a comparative analysis of the associations of individual SNPs alleles and genotypes, their haplotypes, and interlocus interactions in two BMI-distinct groups [BMI < 25; BMI ≥ 25]. To identify correlation of the individual SNPs with EnH_s_ (association parameters [OR, 95%CI] [65,66,67] were calculated in one genetic model (additive)), we used the gPlink program [68]. When performing calculations, covariates were taken into account. After conducting a comparative analysis of the biomedical characteristics of EnH_s_/control in groups with different BMI [BMI < 25; BMI ≥ 25] (data from Table 1), a list of parameters was determined that were included as covariates (characteristics for which differences were found between EnH_s_ and control) when identifying SNP-disease associations. So, the covariates in both groups under consideration were such factors as BMI (*p* < 0.001 [BMI ≥ 25]), history of infertility (*p* < 0.01 [BMI < 25; BMI ≥ 25]), family history of uterus benign proliferative disorder (*p* < 0.001 [BMI < 25; BMI ≥ 25]), chronic endometritis (*p* < 0.01 [BMI < 25] and *p* < 0.001 [BMI ≥ 25]), fewer births (*p* < 0.01 [BMI < 25; BMI ≥ 25]), and higher number of induced abortions (*p* < 0.001 [BMI < 25; BMI ≥ 25]). The problem of multiple comparisons at this stage of the study (assessment of associations of individual SNPs with EnH_s_ in two comparison groups) was solved by introducing an additional Bonferroni correction (equal to the number of pairs of groups being compared, n = 2 [BMI < 25; BMI ≥ 25]), using permutation testing (to correct the number of analyzed SNPs), and analyzing only one of the most commonly accepted genetic models (additive). Thus, the associations of individual SNPs with EnH_s_, which corresponded to a level of p_(perm)_ < 0.025, were considered statistically significant. EnH_s_-associated loci were measured for statistical power by Quanto (v.1.2.4) [70].

When analyzing the associations of haplotypes with EnH_s_ in the two groups of women under consideration [BMI < 25; BMI ≥ 25], two Bonferroni corrections were introduced to solve the problem of multiple comparisons (minimizing false-positive results), taking into account: (a) the number of possible haplotypes for the two analyzed loci, rs11031002-rs11031005 *FSHB* (n = 4); (b) the number of analyzed groups (n = 2, BMI < 25; BMI ≥ 25). We also used permutation testing to confirm the significance of the associations [69]. So, association of haplotypes with EnH_s_ that had a statistical significance level of at least p_(perm)_ ≤ 0.0062 [0.05/4/2] was considered statistically significant.

Two programs such as MB-DDR (v. 2.6) [71] and MDR (v. 3.0.2) [72] were used by us to identify and visualize, respectively, the interlocus (intergenic) interactions involved in EnH_s_ susceptibility. Importantly, for confirmatory permutation testing [68], we selected models that satisfy the following level of statistical significance (two Bonferroni corrections were additionally introduced, taking into account (a) the number of possible combinations of 9 studied loci at different levels of interaction [2, 3 and 4 level interactions] [39,41], and (b) the number of analyzed groups [n = 2, BMI < 25; BMI ≥ 25]. So, models were selected that had a level of statistical significance at least–P_Bonf_ < 0.69 × 10^−3^ [0.05/36/2] (2-level models); P_Bonf_ < 2.97 × 10^−4^ [0.05/84/2] (3-level); or P_Bonf_ < 1.96 × 10^−4^ [0.05/126/2] (4-level). The work included the best models for which p_(perm)_ ≤ 0.001.

### 2.4. EnH_s_-Involved SNP/Gene/Protein Presumptive Functionality

The hypothetical functionality of EnH_s_-involved loci (taking into account strongly coupled variants at r^2^ ≥ 0.80 [73] and EnH_s_-related biological pathways) were studied at the final stage of our study in a comparative aspect for groups with BMI < 25 and BMI ≥ 25. To do this, we used in silico methodology [65] and a wide range of modern bioinformatic online databases [73] such as GTExportal (accessed on 13 March 2024) [74]; HaploReg (accessed on 16 March 2024) [59]; Gene Ontology (accessed on 19 March 2024) [75]; PolyPhen2 (accessed on 22 March 2024) [76]; STRING (accessed on 25 March 2024) [77]; and SIFT (accessed on 29 March 2024) [78].

## 3. Results

In the studied groups of women (EnH_s_, control) with different BMI (BMI < 25; BMI ≥ 25), the distribution of analyzed polymorphisms obeyed the Hardy–Weinberg law (P_bonferroni_ > 0.05/2 group/9 SNPs = 0.00278) (Tables S3 and S4).

In general, the studied sample (EnH_s_ [n = 520]/control [n = 973], n = 1493) revealed an association of interactions of rs148982377 [T>C] *ZNF789* × BMI with EnH_s_: OR = 1.11, 95%CI = 1.02–1.22, *p* = 0.019, p_(perm)_ = 0.024 (Table 2).

The analysis of SNP associations with EnH_s_ among women with a BMI < 25 showed the involvement of two polymorphic loci in the development of the disease, including rs11031002 [T>A] *FSHB* (OR = 0.46; 95%CI = 0.28–0.74; *p* = 0.002; p_(perm)_ = 0.002; power = 94.75%) and rs11031005 [T>C] *FSHB* (OR = 0.58; 95%CI = 0.39–0.93; *p* = 0.024; p_(perm)_ = 0.025; power = 78.93%) (Table 2). The EnH_s_ risk value of the TT*rs11031002-rs11031005 *FSHB* haplotype was revealed (OR = 2.31; *p* = 0.0004; p_(perm)_ = 0.003) (Table 3).

In women with a BMI ≥ 25, the three polymorphisms of sex hormone genes such as rs11031002 [T>A] *FSHB* (OR = 0.43, 95%CI = 0.29–0.65, *p* = 0.00005, p_(perm)_ = 0.00007; power = 99.11%), rs11031005 [T>C] *FSHB* (OR = 0.46, 95%CI = 0.32–0.68, *p* = 0.00009, p_(perm)_ = 0.0002, power = 98.25%), and rs148982377 [T>C] *ZNF789* (OR = 1.77, 95%CI = 1.11–2.84, *p* = 0.017, p_(perm)_ = 0.023, power = 77.78%) were EnH_s_-associated (Table 2). Statistically reliable data were obtained on the association of haplotypes of the rs11031002–rs11031005 haploblock of the *FSHB* gene with EnH_s_ formation: the TT*rs11031002–rs11031005 haplotype showed associations with the disease (OR = 3.27; *p* = 4 × 10^−9^; p_(perm)_ = 1 × 10^−6^).

Thus, in this section of the work, as a result of a comparative analysis, we have established both common protective factors for EnH_s_ in women with a BMI ≥ 25 and BMI < 25 (rs11031002 [T>A] and rs11031005 [T>C] *FSHB*) and a specific risk factor for the development of the disease in individuals with a BMI ≥ 25 (rs148982377 [T>C] *ZNF789* was associated with EnH_s_ in women with a BMI ≥ 25 (OR = 1.77) and was not linked with the disease in women with a BMI < 25). The unidirectional effects of minor alleles of polymorphisms, which are common protective factors for the development of the disease in women with different BMI, are noteworthy: the alleles A rs11031002 *FSHB* and C rs11031005 *FSHB* have a protective value in the occurrence of the disease in women with a BMI ≥ 25 and a BMI < 25. Their reference alleles in the haplotype (TT*rs11031002-rs11031005 *FSHB*) were associated with a significantly higher risk of disease formation in both women with a BMI ≥ 25 (OR = 3.27) and women with a BMI < 25 (OR = 2.31), and at the same time, as can be seen from the above OR indicators, the risk effect of this haplotype in women with a BMI ≥ 25 is more pronounced (by 41%) than in women with a BMI < 25.

As a result of statistical modeling, only 3 models of 4 SNPs interactions (rs34670419 [G>T] *ZKSCAN5*, rs11031002 [T>A], rs11031005 [T>C] *FSHB*, and rs117585797 [C>A] *ANO2*) of three levels (one model for each level) associated with EnH_s_ were obtained in women with a BMI < 25 at a p_(perm)_ ≤ 0.001 (Table 4), in which only six specific combinations of genotypes (two at each level) were disorder-associated (Table S5). The two-locus interaction rs11031002 [T>A] *FSHB* × rs11031005 [T>C] *FSHB* is the basis of all three most significant models. The highest level of statistical significance and the highest degree of severity of the correlation with EnH_s_ was recorded for two protective combinations of genotypes, including two (rs11031002 TA × rs11031005 TT; *beta* = −3.36, *p* = 0.00004) and three (rs11031002 TA × rs117585797 CC × rs11031005 TT; *beta* = −3.36, *p* = 0.00004) loci (Table S5).

**Figure 1:**
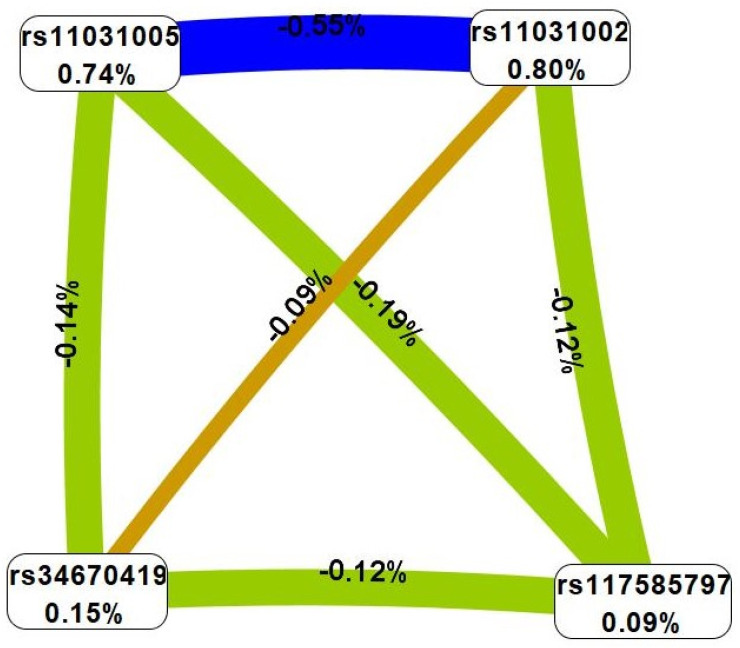
The entropy graph of the SNP × SNP interactions with simple endometrial hyperplasia among BMI < 25 female based on the MDR analysis. Positive values of entropy indicate synergistic interactions while the negative values indicate redundancy. The red and orange colors denote strong and moderate synergism, respectively; brown denotes the independent effect; and green and blue denote moderate and strong antagonism.

The constructed interlocus interactions graph of all 4 SNPs linked with EnH_s_ in women with a BMI < 25 (as part of haplotypes and intergenic interactions models) revealed further pronounced antagonistic interactions between polymorphisms rs11031005 [T>C] *FSHB* and rs11031002 [T>A] *FSHB* (determines −0.55% of the disorder variance) (Figure 1).

The results obtained during the modeling of intergenic interactions that determine EnH_s_ susceptibility in women with a BMI of ≥25 are presented in Table 4. We established 12 interlocus interaction models covering all studied three levels (2, 3 and 4 SNPs), including 7 loci of sex hormone genes (rs148982377 [T>C] *ZNF789*, rs34670419 [G>T] *ZKSCAN5*, rs11031002 [T>A], rs11031005 [T>C] *FSHB*, rs112295236 [C>G] *SLC22A10*, rs117585797 [C>A] *ANO2*, and rs117145500 [A>C] *CHD9*), associated with the EnH_s_ risk at p_(perm)_ < 0.001. Half of these models (6/12) were four-locus. Almost all models included two polymorphisms of the *FSHB* gene—rs11031002 [T>A] (12/12, 100%) and rs11031005 [T>C] (11/12, 91.67%)—and the epistatic interaction of these two loci was the basis of 91.67% of all the most significant models (11/12, 91.67%). The majority of EnH_s_-significant polymorphisms (4 SNPs: rs148982377 [T>C] *ZNF789*, rs34670419 [G>T] *ZKSCAN5*, rs112295236 [C>G] *SLC22A10*, rs117585797 [C>A] *ANO2*) were an integral part of 4 models each (33.33%). In total, 42 different multi-level combinations of genotypes were obtained (5 [11.90%]—2 loci, 16 [38.10%]—3 loci, 21 [50.00%]—4 loci), determining the risk of developing EnH_s_ in women with a BMI ≥ 25 (Table S6), among which a majority (26/42, 61.90%) had a protective value in relation to the disease, and a minority (16/42, 38.10%) was at risk. Four protective combinations of genotypes demonstrated the highest level of statistical significance of association with EnH_s_, and for two of them this parameter exceeded the value accepted as a standard in GWAS (*p* = 5 × 10^−8^): rs11031002*TT × rs11031005*TT (*beta* = 1.17, *p* = 6 × 10^−9^), rs11031002*TT × rs117585797*CC × rs11031005*TT (*beta* = 1.04, *p* = 5 × 10^−8^), rs11031002*TT × rs112295236*CC × rs11031005*TT (*beta* = 0.92, *p* = 2 × 10^−7^), rs11031002*TT × rs117585797*CC × rs112295236*CC × rs11031005*TT (*beta* = 0.87, *p* = 4 × 10^−7^) (Table S6).

**Figure 2:**
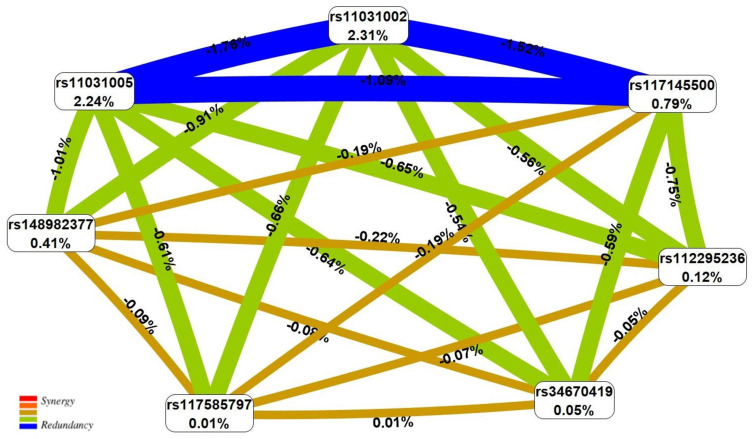
The entropy graph of the SNP × SNP interactions with simple endometrial hyperplasia among BMI ≥ 25 female based on the MDR analysis. Positive values of entropy indicate synergistic interactions while the negative values indicate redundancy. The red and orange colors denote strong and moderate synergism, respectively; brown denotes the independent effect; and green and blue denote moderate and strong antagonism.

The results of qualitative (nature of interaction) and quantitative (percentage of entropy of the disease) assessment of the contribution of polymorphic loci and their epistatic interactions to EnH_s_ susceptibility in women with a BMI ≥ 25, in the form of graphs, are presented in Figure 2. We found pronounced antagonistic interactions at the level of −1.09–−1.76% between the three loci—rs11031002 [T>A], rs11031005 [T>C] *FSHB*, and rs117145500 [A>C] *CHD9* (shown in blue in the figures)—and expressed independent effects of rs11031002 [T>A] (2.31%) and rs11031005 [T>C] (2.24%) of the *FSHB* gene. Epistatic interactions of SNP rs148982377 [T>C] *ZNF789* (the main effect in the disease formation in women with a BMI ≥ 25) with rs11031002 [T>A] and rs11031005 [T>C] *FSHB* also have an antagonistic orientation (moderate antagonism, indicated in green in the Figure 2) and affect −0.91–−1.01% of the EnH_s_ variance in the study group of women.

Thus, we have established a greatly more significant contribution of the interlocus interactions of the considered sex hormone candidate genes to EnH_s_ formation in women with BMI ≥ 25 (7 SNPs in 12 models) compared with women with BMI < 25 (4 SNPs in 3 models), with approximately the same volumes of studied samples in these groups (n = 766 [BMI < 25] and n = 727 [BMI ≥ 25]) and the same threshold level of selection of the most significant models (p_(perm)_ ≤ 0.001).

### 3.1. Presumptive Functionality of Loci Involved in Susceptibility to EnH_s_ in Women with Different BMI

In this section of the work, the hypothetical functional significance of SNPs was assessed, which showed an association with the developing EnH_s_ in women with different BMI: 7 SNPs in individuals with a BMI ≥ 25 (rs11031002 [T>A] and rs11031005 [T>C] *FSHB*, rs148982377 [T>C] *ZNF789,* rs34670419 [G>T] *ZKSCAN5*, rs112295236 [C>G] *SLC22A10*, rs117585797 [C>A] *ANO2*, rs117145500 [A>C] *CHD9*) and 4 SNPs in individuals with BMI < 25 (rs11031002 [T>A] and rs11031005 [T>C] *FSHB*, rs34670419 [G>T] *ZKSCAN5*, rs117585797 [C>A] *ANO2*) in the body as a whole, with an emphasis on adipose tissue.

#### 3.1.1. Epigenetic Changes

Among women with a BMI ≥ 25, seven SNPs associated with EnH_s_ and 76 strongly linked loci have regulatory effects on 9 different genes—CHD9, ANO2, RP11-467J12.4, FSHB, SLC22A24, SLC22A10, SLC22A25, ZKSCAN5, and ZNF789 (Table S7). In the group of women with a BMI < 25, four SNPs associated with EnH_s_ and 4 proxy variants have regulatory effects on 4 different genes—ANO2, FSHB, ZKSCAN5, and ZNF789 (Table S7).

Interestingly, hypothetical significant regulatory effects (located in the region of promoters, enhancers, active promoters) in various cell lines of adipose tissue (mesenchymal stem cells, which are precursors of adipocytes, fat cells, etc.) exhibit polymorphic loci that demonstrate the main effects in EnH_s_ formation in women with a BMI ≥ 25 (rs148982377 [T>C] *ZNF789*, OR = 1.77; strongly coupled SNP rs34670419 [G>T] *ZKSCAN5*) (Table 5). The SNP rs112295236 [C>G] *SLC22A10* (and its strongly linked loci) exhibit significant epigenetic effects (they were localized in the area of potential enhancers) in the region of *SLC22A10*, *SLC22A24*, and *SLC22A25* genes in adipose tissue (adipose derived mesenchymal stem cell cultured cells). The impact epigenetic effects in adipose tissue of polymorphisms located in the region of the genes *ZKSCAN5* and *ZNF789* (rs148982377 [T>C] *ZNF789* and rs34670419 [G>T] *ZKSCAN5*) identified by us in silico may be one of the biomedical justifications for the involvement of SNPs of sex hormone genes in EnH_s_ formation in women with different BMI.

#### 3.1.2. Regulatory Influences on Gene Expression (eQTL)

Among women with a BMI ≥ 25, five EnH_s_-causal SNPs and 61 strongly linked loci are associated with the transcription level of 10 different genes—*CYP3A7*, *ATL3*, *GS1-259H13.2*, *OR2AE1*, *PTCD1*, *TRIM4*, *ZKSCAN5*, *ARL14EP*, *SLC22A10* and *SLC22A9* (Tables S8 and S9). In the group of women with a BMI < 25, four EnH_s_-associated SNPs and three proxy variants also affected the expression of 7 genes (*CYP3A7*, *GS1-259H13.2*, *OR2AE1*, *PTCD1*, *TRIM4*, *ZKSCAN5*, *ARL14EP*) (Tables S8 and S9).

Interestingly, four EnH_s_-correlated polymorphic loci were eQTL impact in adipose tissue—rs11031002 [T>A] and rs11031005 [T>C] *FSHB*, rs148982377 [T>C] *ZNF789*, rs34670419 [G>T] *ZKSCAN5* (Table S8)—and two strongly linked to rs11031005 [T>C] *FSHB* SNPs (rs11031006, rs74485684) (Table S9). Thus, minor allelic variants A rs11031002 and C rs11031005 *FSHB* (protective factors for EnH_s_ development in both women with a BMI ≥ 25 and BMI < 25) were associated with higher *ARL14EP* transcription in subcutaneous adipose tissue (NES = 0.25; *p* = 2.9 × 10^−6^ and NES = 0.25; *p* = 5.3 × 10^−6^ respectively) (Table S8). Two SNPs which were in linkage disequilibrium (LD) with the rs11031005 [T>C] *FSHB* SNPs, rs11031006 and rs74485684, were also correlated with the expression level of the same gene in subcutaneous adipose tissue (Table S9). The minor allele C rs148982377 *ZNF789* (the main risk factor for EnH_s_ development in women with a BMI ≥ 25) was associated with increased transcriptional activity of the *CYP3A7* gene in visceral adipose tissue (NES = 0.74; *p* = 1.7 × 10^−7^) (Table S8). The expression of the same gene in visceral adipose tissue was also influenced by rs34670419 [G>T] *ZKSCAN5* (Table S9), which was associated with EnH_s_ within the framework of interlocus interactions and at the same time was in disequilibrium with rs148982377 [T>C] *ZNF789* (Table S9).

#### 3.1.3. Regulatory Influences on Gene Splicing (sQTL)

Two polymorphisms involved in the formation of susceptibility to EnH_s_ in women with different BMI—rs148982377 [T>C] *ZNF789* (the main risk factor for EnH_s_ development in women with a BMI ≥ 25) and rs34670419 [G>T] *ZKSCAN5* (associated with the disease in both BMI groups as part of interlocus interactions) were associated with the level of *GPC2* alternative splicing (Tables S10 and S11).

So, it can be summarized that 7 SNPs and 76 proxy loci associated with the risk of developing EnH_s_ in women with a BMI ≥ 25 were presumptively functionally significant for 18 genes (*CHD9*, *RP11-467J12.4*, *ANO2*, *FSHB*, *ZKSCAN5*, *ZNF789*, *CYP3A7*, *GPC2*, *GS1-259H13.2*, *OR2AE1*, *PTCD1*, *TRIM4*, *ARL14EP*, *ATL3*, *SLC22A10*, *SLC22A9*, *SLC22A24*, *SLC22A25*) and 12 regulatory proteins (FOXA1, FOXA2, CFOS, P300, GATA2, HDAC2, TCF4, MAFF, CEBPB, RXRA, SP1, MAFK). Also, 4 SNPs and 4 LD variants correlated with EnH_s_ in women with a BMI < 25 were presumptively functionally significant for 11 genes (*ANO2*, *FSHB*, *ZKSCAN5*, *ZNF789*, *CYP3A7*, *GPC2*, *GS1-259H13.2*, *OR2AE1*, *PTCD1*, *TRIM4*, *ARL14EP*).

At the same time, the loci associated with EnH_s_ in women with different BMI showed their functionality in relation to 7 genes (*ZKSCAN5*, *ZNF789*, *SLC22A24*, *SLC22A10*, *SLC22A25*, *CYP3A7*, *ARL14EP*) in adipose tissue. Based on these data, at the next stage of our work, an assessment of protein interactions and their biological pathways was carried out in two directions: (a) in a comparative aspect for women with BMI < 25 and BMI ≥ 25; (b) for genes whose functionality in adipose tissue depends on EnH_s_-significant loci.

#### 3.1.4. Biological Pathways of EnH_s_-Involved Protein Interactions

**Figure 3:**
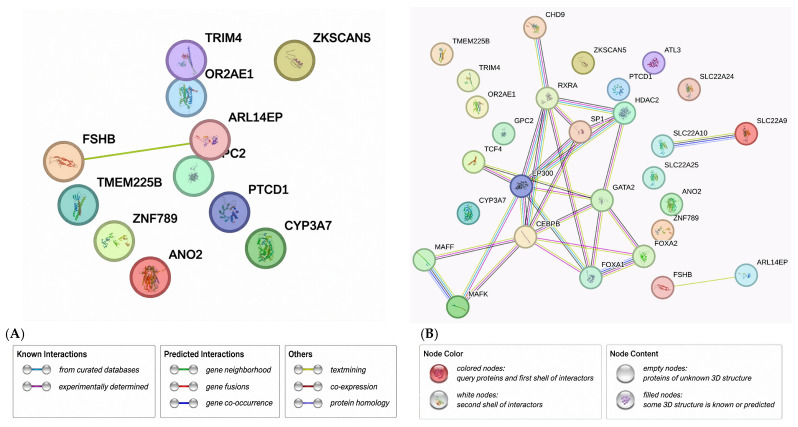
A network of protein interactions associated with EnH_s_ in women with a BMI < 25 (**A**) and women with a BMI ≥ 25 (**B**) (STRING data).

Using the STRING program, we evaluated the interaction of 11 proteins involved in the formation of EnH_s_ in women with BMI < 25 (all 11 were encoded by genes functionally associated with 8 loci [4 EnH_s_-related loci and 4 LD SNPs]) and 30 proteins involved in the formation of EnH_s_ in women with BMI ≥ 25 (18 were encoded by genes functionally correlated with 83 polymorphisms (7 EnH_s_-causal loci and 76 LD SNPs) and 12 regulatory proteins). The results are presented in Figure 3.

Based on the information presented in Figure 3, we can state significant differences in the interaction of proteins (and their involvement in biological pathways) involved in the formation of EnH_s_ in the groups of women with BMI < 25 and BMI ≥ 25 under consideration. Thus, among women with a BMI < 25, EnH_s_-impact protein interactions were rather insignificant (only interactions of FSHB with ARL14EP were recorded, score = 0.474) (Figure 3A) and they were involved according to the Monarch database in the regulation of dehydroepiandrosterone sulphate (EFO:0007001; *p* = 0.0018) (PTCD1, ZNF789, ZKSCAN5) and progesterone (EFO:0007004; *p* = 0.0481) (ZNF789, ZKSCAN5) levels. On the contrary, in women with a BMI ≥ 25, EnH_s_-impact protein interactions were more pronounced (the role of hub-proteins was performed by CHD9, FOXA1, FOXA2,300, GATA2, HDAC2, TCF4, MAFF, CEBPB, RXRA, SP1, MAFK) (Figure 3B) and they were involved in numerous mechanisms of transcription regulation: regulation of transcription by RNA polymerase II (GO: 0006357; *p* = 0.0134) (FOXA1, EP300, CEBPB, SP1, ZNF789, TCF4, ZKSCAN5, MAFK, GATA2, FOXA2, FSHB, RXRA, HDAC2, MAFF), RNA polymerase II *cis*-regulatory region sequence-specific DNA binding (GO: 0000978; *p* = 0.0011) (FOXA1, CEBPB, SP1, ZNF789, MAFK, GATA2, ZKSCAN5, TCF4, FOXA2, RXRA, MAFF), RNA polymerase II-specific DNA-binding transcription factor binding (GO:0061629; *p* = 0.0031) (EP300, CEBPB, SP1, GATA2, RXRA, HDAC2), transcription factor binding (GO: 0008134; *p* = 0.0040) (EP300, CEBPB, SP1, GATA2, TCF4, RXRA, HDAC2), transcription co-regulator binding (GO: 0001221; *p* = 0.0063) (EP300, SP1, GATA2, RXRA], etc. It is important to note the presence of adipose-specific biological pathways in the protein interactions in women with a BMI ≥ 25 (shown in Figure 3B): adipogenesis (WP236; *p* = 0.0096) (CEBPB, SP1, GATA2, RXRA), transcriptional cascade regulating adipogenesis (WP4211; *p* = 0.0263) (CEBPB, GATA2), and transcriptional regulation of white adipocyte differentiation (HSA-381340; *p* = 0.0040 (EP300, CEBPB, CHD9, RXRA). Interestingly, according to the KEGG database, the protein interactions under consideration in women with a BMI ≥ 25 were significant for such a biological pathway as transcriptional misregulation in cancer (hsa05202; *p* = 0.0349) (CEBPB, SP1, RXRA, HDAC2).

**Figure 4:**
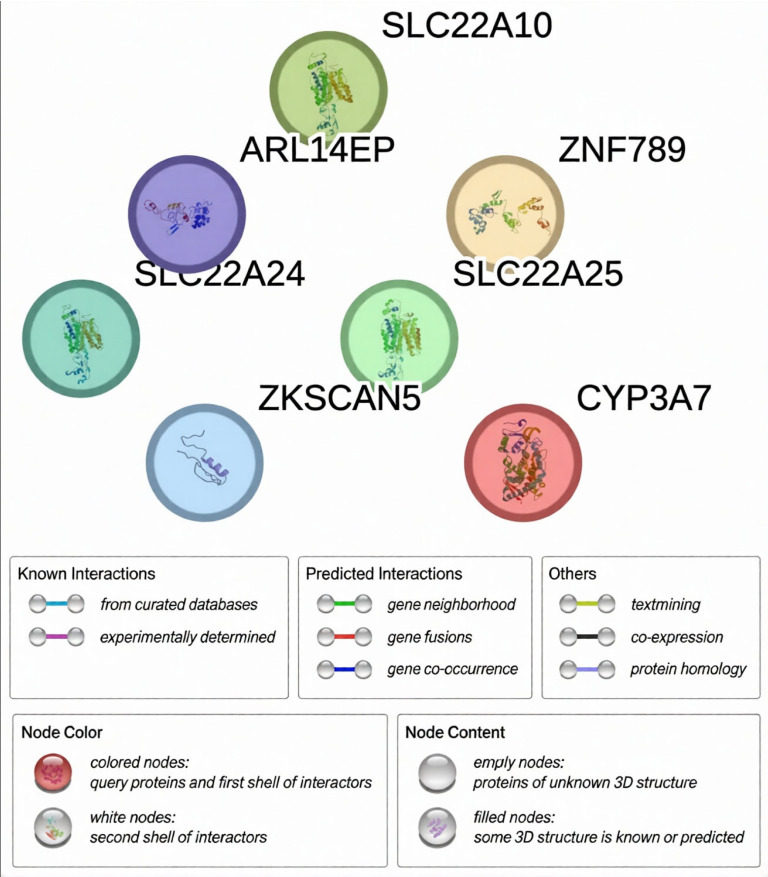
A protein-interactions network whose genes were functionally significant in adipose tissue and depend on EnH_s_-associated interactions (STRING data).

When studying the interaction of proteins whose genes were functionally significant in adipose tissue and depend on loci associated with EnH_s_ development, no pronounced interactions were revealed (Figure 4). According to the Monarch database, these proteins affect progesterone measurement (EFO:0007004; *p* = 0.0369) (ZNF789, ZKSCAN5), and according to Protein Domains and Features (InterPro) materials, they interact due to major facilitator superfamily domain (IPR0208463; *p* = 0.0314) (SLC22A25, SLC22A10, SLC22A24) and MFS transporter superfamily (IPR036259; *p* = 0.0451) (SLC22A25, SLC22A10, SLC22A24).

## 4. Discussion

In the studied sample (EnHs/control, n = 1493), an association of interactions of rs148982377 [T>C] *ZNF789* × BMI with EnHs (OR = 1.11) was revealed. As a result of a comparative analysis, we found that rs148982377 [T>C] *ZNF789* was associated with EnH_s_ among women with a BMI ≥ 25 (OR = 1.77) and was not linked with the disease in women with a BMI < 25. Both common protective factors for EnH_s_ were also identified in women with BMI ≥ 25 and BMI < 25 (rs11031002 [T>A] *FSHB* (OR = 0.43 and OR = 0.46 respectively) and rs11031005 [T>C] *FSHB* (OR = 0.46 and OR = 0.58 respectively)). At the same time, in women with a BMI ≥ 25, the risk effect of the TT*rs11031002–rs11031005 haplotype (OR = 3.27) is more pronounced (by 41%) and the contribution of interlocus interactions of sex hormone genes to EnH_s_ formation was more significant (7 SNPs in 12 models) than in women with a BMI < 25 (OR = 2.31 and 4 SNPs in 3 models respectively).

In the sample we studied, the BMI in patients with EnH_s_ (26.94 ± 5.56) was significantly higher (*p* < 0.001) than in the control (25.22 ± 4.52), overweight/obesity significantly increased the risk of developing EnH_s_ (OR = 2.34 95%CI 1.86–2.93 *p* = 0.005), and overweight/obesity, according to the data obtained in this work, were significant modifiers of the involvement of SNPs of sex hormone genes in the disorder formation. The association of overweight and obesity with EnH_s_ risk has been indicated in numerous studies [9,10,79]. In the work of Epplein et al., a sample of 892 women (an equal number of patients with EnH_s_ and controls were studied) convincingly showed a significant increase in disease risk in women younger than 52 years with an increase in BMI: compared with women who were not overweight (BMI ≤ 24.9), the EnH_s_ risk with atypia increased 2.3-fold (BMI 25–29.9), 3.7-fold (BMI 30–39.9), or 13.0-fold (BMI ≥ 40), and the risk of complex EnH_s_ increased 3.0-fold (BMI 25–29.9), 4.6-fold (BMI 30–39.9), or 23.0-fold (BMI ≥ 40) [9]. A retrospective analysis of 916 premenopausal women with abnormal uterine bleeding showed that patients with a BMI>30 developed complex EnH_s_ or endometrial cancer 4 times more often than women with a lower BMI [10].

Overweight and obesity cause an increased level of estrogens in the female organism (relative to progesterone), which is risky for the occurrence of EnH_s_, and this may be based on a number of biological mechanisms [5,9,79,80]. Firstly, the secretory activity of the adrenal glands increases, which leads to an increase in the number of androgen precursors, which can then be converted to estradiol in peripheral tissues (mainly adipose) [5].

Secondly, the conversion of androgens (androstenedione) into estrogens (estrone) increases in excess adipose tissue [5,9,79]. Estrogens, interacting with their specific receptors, through various genomic mechanisms (due to changes in the transcriptional activity of targeted genes [estrogen-dependent genes], whose protein products are involved in the processes of growth, differentiation, angiogenesis, and apoptosis,) and non-genomic biological pathways (due to interaction with various proteins such as adapter proteins, G-class proteins, cytoplasmic kinases (PI3K, MAPK, AKT), growth factor receptors (IGFR1,EGFR, etc.), signaling enzymes (adenyl cyclase), etc.) [81] affect the proliferative activity of cells, including endometrial cells [5,9,79].

Thirdly, the SHBG content, which is a key regulator of free estrogens and testosterone levels, decreases, which leads to an increase in the concentration of biologically active forms of these sex hormones [79]. A direct, pronounced association of BMI (overweight/obesity) with estrogens and androgens and an inverse correlation with SHBG have been convincingly shown in numerous studies, including those performed by the method of Mendelian randomization of GWAS data [82,83,84]. At the same time, a decrease in BMI in women caused a decrease in the concentration of estrogens and testosterone and an increase in SHBG levels [85,86], while an increase in BMI led to a significant increase in estradiol levels (by 45–68%), estrone (21–34%), free forms of estradiol (101%) and testosterone (35%), as well as a decrease in the concentration of SHBG (by 6–35%) [87], which certainly may be of paramount importance in EnH_s_ pathophysiology.

Fourth, various disorders of folliculogenesis are formed and anovulation develops [5,76,88,89]. In obese women compared with non-obese individuals, during a prospective longitudinal study of 42 subjects, significant changes were recorded at various stages of folliculogenesis: reduced follicle recruitment, fewer progressive follicles, a reduced number of anovulatory follicles that have achieved dominance, a smaller diameter of ovulatory follicles, and luteal phase defects associated with low progesterone levels [89]. In the work of Yeung et al., among obese women, compared with individuals with normal body weight, lower levels of progesterone (−15%), LH (−17%) and FSH (−23%), higher concentrations of free estradiol (+22%), and large changes in the amplitude of LH and FSH throughout the cycle were revealed. Changes in the levels of free estradiol, LH and FSH, but with a lesser degree of severity, were also observed in overweight women (BMI = 25–30) [88]. It is indicated that with an average age of manifestation of EnH with atypia of more than 40 years, in women with chronic amenorrhea (chronic anovulation), the EnH risk increases significantly already at the age of 20–30 years [80]. A sample of 1200 women showed a significant increase in the risk of anovulation in women with an increase in BMI [90].

Ultimately, the above pathophysiological mechanisms cause the formation of a more pronounced imbalance in the estrogens–progesterone system in obese and overweight women (hyperestrogenism with a relative lack of progesterone), which leads to estrogen-induced excessive proliferation of endometrial cells and the appearance of EnH [5,9,79], and can also modify the disease-significant risk effects of hormone-related polymorphic loci, which we established in our study.

In this work it was found that the allele C rs148982377 [T>C] *ZNF789* (OR = 1.77) serves as a specific risk factor for the formation of the disease in women who are overweight/obese. Based on in silico data, we have shown that the allele variant C rs148982377 [T>C] *ZNF789*, which is risky for EnH_s_, was associated with higher expression of the *CYP3A7* gene in visceral adipose tissue. The expression product of the *CYP3A7* gene is the protein of the same name (CYP3A7, cytochrome P450, family 3, subfamily A, member 7), which is a member of the cytochrome P450 enzyme superfamily. The enzyme CYP3A7 participates in the processes of hydroxylation (16α-hydroxylation) of the metabolite of testosterone and DHEAS-estrone to 16α-hydroxyestrone (16α-OHE1) [91,92]. It is noted in the literature that 16α-OHE1 is a highly active estrogenic metabolite, more potent than the widely known estradiol [92]. It is believed that 16α-OHE1, binding to estrogen receptors, exhibits strong mitogenic effects on endometrial cells [93,94], which may explain the links we have identified between the highly productive allele C rs148982377 [T>C] *ZNF789* with respect to the *CYP3A7* gene and an increased risk of developing EnH_s_ in overweight/obese women. It is important to note that with overweight/obesity, the processes of peripheral conversion of androgens into estrogens in adipose tissue are much more intense [94] and therefore, in obese women, more pronounced pathological effects can be expected (increased risks of developing EnH, endometrial cancer, etc.) increased levels of estrogens and their active metabolites (16α-OHE1, etc.) [4,5,95,96].

So, the results obtained by us in silico and the literature data on this issue suggest a possible pathophysiological justification for the associations of rs148982377 [T>C] *ZNF789* identified by us with the EnH_s_ risk (OR = 1.77): “increased expression of *CYP3A7* in adipose tissue in overweight/obese women with the allele C rs148982377 [T>C] *ZNF789* → increased highly active metabolite of estrone—16α-OHE1, under the action of overly synthesized CYP3A7 → more pronounced mitogenic effects of 16α-OHE1 on the endometrium → increased risk of developing EnH_s_”!

There are indications in the literature (although contradictory) of a link between the level of CYP3A7 production and endometrial cancer [95,96,97]. In an experimental paper by Fishman et al. it has been shown that in women with breast and endometrial cancer, compared with controls, 16α-hydroxylation was significantly increased [95]. The authors note that an increased risk of breast and endometrial cancer may be mediated by one of the products of 16α-hydroxylation—16α-OHE1 [95]. The risk value of 16α-OHE1 in endometrial cancer in postmenopausal women is also shown in the work of Zeleniuch-Jacquotte et al. [96]. At the same time, in a study by Hevir et al. a lower level of CYP3A7 was found in the endometrium of patients with endometrial cancer [97]. There are studies that have not revealed the association of 16α-OHE1 with endometrial cancer in postmenopausal women [98,99].

Four different studies presented in the GWAS catalog show the relationship of polymorphism rs148982377 [T>C] *ZNF789* with the level of androgens and their metabolites, as well as a wide range of other metabolites: DHEAS [16], bioavailable testosterone (in premenopausal women) [20], 6α-hydroxy-DHEAS [100], DHEAS, androsterone sulfate, epiandrosterone sulfate, (3β, 17β) androstenediol disulfate, (3α, 17α) androstenediol monosulfate, X-21470, X-26109, X-24947, etc. [23]. At the same time, the allelic variant C (according to our data, it has an EnH_s_ risk value in overweight/obese women) was associated with lower levels of androgens and their metabolites. In another GWAS, an association of SNP rs148982377 [T>C] *ZNF789* with the DNA repair protein RAD51 (homologue 4) was found [101]. In the work of Diener et al., associations of rs148982377 [T>C] *ZNF789* with nine different metabolites of steroid hormones, mainly DHEAS and androsterone conjugates, have also been demonstrated [102].

Located at a distance of 55.8 kb from the EnH_s_-causal locus rs148982377 [T>C] *ZNF789* and strongly linked to it, the SNP rs34670419 *ZKSCAN5* in a number of GWAS has shown significant associations with progesterone levels [16], DHEAS and cortisol ratio/DHEAS [15], and 4-androsten-3beta,17beta-diol disulfate, 4-androsten-3beta,17beta-diol monosulfate levels [21]; it is also correlated with the risk of endometrial cancer [103] and bone mineral density [104]. Two more SNPs were associated with the metabolite X-21410 and androsterone sulfate, which were in LD with rs148982377 [T>C] *ZNF789*—rs12535424 (r^2^ = 0.67/D’ = 1.00) [21] and rs13243267 (r^2^ = 0.67/D’ = 1.00) respectively [105].

Based on the above literature data, it can be concluded that the allele C rs148982377 [T>C] *ZNF789*, which is a risk factor for EnH_s_ formation in overweight/obese women (OR = 1.77), determines the low level of various androgens and their metabolites in the organism. It can be assumed that relatively low levels of androgens in women with overweight adipose tissue do not provide the protective effects of testosterone at the proper level (as opposed to the proliferative effects of estrogens [13]) in relation to EnH_s_, and consequently the processes of folliculogenesis are disrupted both at an early stage (oocyte metabolism, growth/maturation of primordial follicles, etc.) and at late stages (insufficient inhibition of estrogen formation; impaired corpus luteum formation and progesterone production; insufficient inhibition of endometrial proliferative activity, etc.) [13,106,107,108,109,110,111,112,113,114].

So, in this study, a difference in the nature of association of testosterone-determining polymorphism in women with different BMI was found: testosterone-reducing allele C rs148982377 [T>C] *ZNF789* (but at the same time increasing the level of active metabolites of estrogens, 16α-OHE1) increases the disorder risk in women with a BMI ≥ 25 (OR = 1.77), and it was not associated with the disease in women with a BMI < 25. The data on the nature of testosterone associations with proliferative activity of endometrial cells (including the EnH risk [risk/protective value]) available in numerous literature sources [12,13,90,106,107,108,109,110,111,112,113,114] are multidirectional, and the inconsistency of these relationships and their biomedical justifications persists at the present time despite a large number of experimental works conducted by various scientific teams on this topic [13]. On the one hand, data were obtained on the stimulating effect of testosterone/DHT on endometrial cell proliferation in ARCO mouse models [115], rodents undergoing ovariectomy [116,117], and female transsexuals treated with testosterone [118]; higher concentrations of androgens (as well as estradiol) were detected in patients with adenomatous EnH compared with the control [119]; and the risk of EnH development in such a hyperandrogenic disease as polycystic ovary was increased (due to its characteristic anovulatory cycles) [3,4]. On the other hand, there are numerous experimental/clinical results indicating the antiproliferative effect of androgens on endometrial cells [12,13,120,121,122,123,124,125]. Thus, experimental studies (in vitro) on endometrial cell lines [106,107,108,109,110,111,112,113] and isolated endometrial epithelial cells [126] have shown the inhibitory effect of androgens (androstenedione/testosterone/DHT) on cell proliferation, in contrast to the proliferative effect of estrogens [13]. Among the clinical evidence of the antiproliferative effect of androgens on the uterine endometrium, the results of numerous studies on the use of a synthetic androgen, danazol, in the treatment of EnH can be cited [121,122,123,124,125]. At the same time, it should be noted that the above-mentioned studies, which indicate both antiproliferative and endometrial cell proliferation-stimulating effects of testosterone, are characterized by an extremely pronounced diversity, both in the studied research objects (genetically modified mice, rodents undergoing ovariectomy, female transsexuals, EnH patients, etc.) and the research methodology/techniques used in this work (comparative analysis of androgens in patients/controls, the use of androgens in the treatment of EnH patients and female transsexuals, the study of phenotypic effects in experimental animals undergoing ovariectomy or having knockouts of individual genes, etc.). This makes it significantly difficult to identify any patterns/factors that explain to one degree or another such markedly different (opposite-directional) effects of androgens found in these studies. There is an obvious need to continue experimental research on this topic, the results of which would allow us to conduct sufficiently powerful meta-analyses on this issue and make it possible to identify possible causes/factors/mechanisms underlying such contradictory results. There are isolated studies in the literature indicating differences in the biological effects of testosterone in women with different BMI, including such a testosterone/BMI-related risk factor for EnH development as anovulation [90]. After analyzing the results of observation of 1200 women, the authors found an increased risk of anovulation with an increase in BMI (OR = 1.03) (while taking into account adjustments for the content of free testosterone in the blood serum, lipids, and other factors) [90]. Based on the data obtained, the authors conclude that in women with regular menstruation, anovulation may be independently associated with both testosterone levels and obesity [90]. At the same time, genetic studies that examine the role of factors (polymorphisms) that determine the level of testosterone in the body in EnH_s_ formation have not yet been performed, and therefore we do not have the opportunity to conduct a correct comparative genetic analysis on this topic. Further more-detailed genetic and epidemiological studies, including experimental work on functional genomics and proteomics, are needed to identify additional factors (environmental, genetic) that determine the features of the relationship between BMI, androgens, and EnH_s_, including confirming the modifying effect of overweight/obesity registered in our study on the nature of the association of functionally significant SNPs of sex hormone genes with the risk of developing EnH_s_.

It should be noted that previously performed genetic and epidemiological studies in the population of the Central Chernozem region of Russia under study also showed a significant modifying effect of overweight/obesity on the relationship of a number of candidate genes’ polymorphism with hormone-dependent diseases such as breast cancer (gene SNPs of matrix metalloproteinases and SHBG) [42,43], knee osteoarthritis (SNP of GWAS-significant genes for osteoarthritis of the knee joint) [45], preeclampsia (SNP of GWAS-significant genes for hypertension) [44], and uterine fibroids (SHBG gene SNPs) [46], which suggests a significant role of such an environmental risk factor for hormone-dependent diseases such as overweight/obesity (BMI) in the epigenetic regulation of the phenotypic effects of functionally significant polymorphism in these diseases. A detailed study of the mechanisms (including genetic ones) of BMI-dependent modification of the phenotypic manifestation of hereditary risk factors in various diseases will allow a deeper understanding of the pathophysiology of these disorders.

This study identified common genetic risk/protection factors for EnH_s_ development in women with different BMI, which are polymorphisms rs11031002 [T>A] and rs11031005 [T>C] *FSHB*. Minor alleles of these SNPs reduced the disease risk in women with a BMI < 25 (OR = 0.46 and OR = 0.58 respectively) and a BMI ≥ 25 (OR = 0.43 and OR = 0.46 respectively), and reference alleles of these loci (as part of the TT*rs11031002-rs11031005 haplotype) increased the EnH_s_ risk (OR = 2.31 [BMI < 25] and OR = 3.27 [BMI ≥ 25]). GWAS data indicate the relationship of rs11031002 [T>A] and rs11031005 [T>C] *FSHB* and LD SNPs (rs11031006; rs10835638; rs11031010; rs12294104) with the content of such important regulators of the organism hormonal status as LH and FSH/FSHB [16,18,27,101,127,128,129,130,131,132,133,134]. An imbalance in the content/ratios of LH and FSH can lead to disturbances in the formation of estrogens and progesterone, as a result of which hyperestrogenism (absolute/relative) may develop with a deficiency of progesterone influences, which increases the EnH_s_ risk [3,4,5]. An EnH-significant consequence of pronounced LH/FSH-mediated disorders may be the occurrence of anovulations [135,136], the persistence of which for a long time (chronic anovulation) can lead to the activation of estrogen-induced endometrial proliferation against the background of a deficiency of antiproliferative progesterone effects, which ultimately contributes to EnH development [4,5].

The present study has a number of limitations: (a) The results of the work were obtained on one ethno-territorial group of women (Russians of Central Russia) and they need to be confirmed (replicated) by an independent cohort (including other ethnic groups), so they can be assessed as preliminary. (b) In this study, data were obtained for only one morphological subtype of EnH (EnH_s_) and further study/confirmation of the identified associative relationships in samples of patients with another histological subtype of EnH—EnH with atypia (intraepithelial neoplasia of the endometrium)—is necessary, since this particular subtype of the disease has a high risk of oncotransformation. It should be noted that EnH_s_ and intraepithelial neoplasia of the endometrium have a specific pathophysiology, and therefore the nature of associative relationships in these two different subtypes of EnH may differ significantly and require a detailed independent study. (c) This work was carried out on two compared subgroups of women (BMI < 25; BMI ≥ 25) using several polymorphisms (9 SNPs), with simultaneous comprehensive association analysis (assessment of associations of individual SNPs; analysis of haplotype relationships; study of inter-loci interactions), which required the introduction of necessary corrections for multiple comparisons at each stage of the study (Bonferroni corrections, permutation testing), which allowed us to obtain sufficiently balanced results (taking into account the possible risks of false-positive and false-negative results). At the same time, this moment is the basis for recognizing the results obtained as research and determines the need for their confirmation in more homogeneous (not requiring the introduction of numerous corrections for multiple comparisons) genetic studies. (d) Women in the control group (who did not have any (clinical, anamnestic, ultrasound) manifestations of diseases of the female reproductive organs) lacked histological confirmation of a normal endometrium, which may lead to some potential misclassification of women in this group and cause some distortion (weakening) of identified associations.

## 5. Conclusions

The presented work is exploratory and demonstrates differences in the nature of the association with EnH_s_ in women with different BMI of a testosterone-determining polymorphism: SNP rs148982377 [T>C] *ZNF789* was associated with EnH_s_ in women with a BMI ≥ 25 and was not linked with the disease in women with a BMI < 25.

## Figures and Tables

**Table 1:** Phenotypic characteristics of the study participants.

Parameters	BMI ≥ 25			BMI < 25		
	Cases $\bar{X}$ ± SD/% (n)	Controls $\bar{X}$ ± SD/% (n)	*p*	Cases $\bar{X}$ ± SD/% (n)	Controls $\bar{X}$ ± SD/% (n)	*p*
*N*	324	403	-	196	570	-
Age, years	45.15 ± 9.32	44.07 ± 8.27	>0.05	36.21 ± 8.62	35.28 ± 8.13	>0.05
BMI, kg/m^2^	30.16 ± 4.48	28.51 ± 3.88	**<0.001**	21.61 ± 1.81	21.42 ± 1.83	>0.05
Family history of benign proliferative diseases of the uterus *	34.26 (111)	19.11 (77)	**<0.001**	30.61 (60)	15.61 (89)	**<0.001**
Married	85.80 (278)	85.86 (346)	>0.05	85.71 (168)	85.96 (490)	>0.05
Smoker (yes)	14.81 (48)	15.14 (61)	>0.05	17.86 (35)	18.42 (105)	>0.05
Drinking alcohol (≥7 drinks per week)	2.78 (9)	1.74 (7)	>0.05	4.08 (8)	4.04 (23)	>0.05
Oral contraceptive use	9.87 (32)	10.17 (41)	>0.05	9.69 (19)	10.00 (57)	>0.05
Age at first oral contraceptive use (mean, years)	23.35 ± 2.37	23.72 ± 2.37	>0.05	23.21 ± 2.31	23.54 ± 2.32	>0.05
Age at menarche and menstrual cycle						
Age at menarche, years	13.14 ± 1.26	13.09 ± 1.23	>0.05	13.44 ± 1.29	13.36 ± 1.27	>0.05
Duration of bleeding menstrual (mean, days)	5.11 ± 1.38	4.94 ± 0.94	>0.05	5.14 ± 1.39	4.97 ± 0.96	>0.05
Menstrual cycle length (mean, days)	27.96 ± 2.16	28.19 ± 2.26	>0.05	27.92 ± 2.13	28.17 ± 2.24	>0.05
Reproductive characteristics						
Age at first birth (mean, years)	21.11 ± 2.35	21.57 ± 3.44	>0.05	21.26 ± 2.35	21.72 ± 3.42	>0.05
No of gravidities (mean)	3.03 ± 2.49	2.63 ± 1.56	>0.05	2.61 ± 2.34	2.23 ± 1.51	>0.05
No of births (mean)	1.32 ± 0.90	1.71 ± 0.68	**<0.01**	1.11 ± 0.82	1.41 ± 0.63	**<0.01**
No of spontaneous abortions (mean)	0.21 ± 0.43	0.22 ± 0.48	>0.05	0.23 ± 0.51	0.23 ± 0.49	>0.05
No of induced abortions (mean)	1.52 ± 1.58	0.88 ± 0.90	**<0.001**	1.21 ± 1.51	0.48 ± 0.91	**<0.001**
No of stillbirths	0.03 ± 0.16	0.02 ± 0.14	>0.05	0.02 ± 0.12	0.01 ± 0.11	>0.05
History of infertility	12.34 (40)	5.21 (21)	**<0.01**	11.22 (22)	5.09 (29)	**<0.01**
Gynecological pathologies						
Cervical disorders	28.70 (93)	28.54 (115)	>0.05	22.96 (45)	22.81 (130)	>0.05
History of sexually transmitted disease	26.23 (85)	26.55 (107)	>0.05	26.53 (52)	27.19 (155)	>0.05
Chronic endometritis	16.05 (52)	7.20 (29)	**<0.001**	10.71 (21)	4.56 (26)	**<0.01**
Chronic inflammation of adnexa	36.11 (117)	34.24 (138)	>0.05	31.12 (61)	30.35 (173)	>0.05
Uterine leiomyoma	58.33 (189)	-	-	40.31 (79)	-	-
Endometriosis	36.73 (119)	-	-	32.65 (64)	-	-
Adenomyosis	21.30 (69)	-	-	19.39 (38)	-	-

Note: *—mother had endometrial hyperplasia, uterine leiomyoma, endometriosis, adenomyosis, *p* values < 0.01 are shown in bold.

**Table 2:** Associations of the SNP-BMI interaction with EnH_s_ in total cohorts of patient/control and associations of the studied polymorphisms with EnH_s_ among BMI < 25 and BMI ≥ 25 females.

Chr	SNP	Minor Allele	Gene	n	SNP-BMI Interaction *					Female with BMI < 25					Female with BMI ≥ 25			
					OR	95%CI		P	n	OR	95%CI		P	n	OR	95%CI		P
						L95	U95				L95	U95				L95	U95	
7	rs148982377	C	*ZNF789*	1451	**1.11**	**1.02**	**1.22**	**0.019**	741	0.68	0.35	1.34	0.268	710	**1.77**	**1.11**	**2.84**	**0.017**
7	rs34670419	T	*ZKSCAN5*	1450	1.06	0.97	1.16	0.210	742	0.74	0.37	1.49	0.403	708	1.30	0.74	2.25	0.361
11	rs11031002	A	*FSHB*	1427	0.99	0.93	1.05	0.739	735	**0.46**	**0.28**	**0.74**	**0.002**	692	**0.43**	**0.29**	**0.65**	**0.00005**
11	rs11031005	C	*FSHB*	1452	0.98	0.93	1.04	0.476	744	**0.58**	**0.39**	**0.93**	**0.024**	708	**0.46**	**0.32**	**0.68**	**0.00009**
11	rs112295236	G	*SLC22A10*	1440	1.02	0.95	1.10	0.570	737	1.66	0.97	2.86	0.065	703	1.05	0.64	1.71	0.845
12	rs117585797	A	*ANO2*	1428	0.99	0.91	1.09	0.952	732	0.66	0.28	1.56	0.343	696	1.10	0.50	2.44	0.814
16	rs117145500	C	*CHD9*	1427	1.02	0.97	1.07	0.489	733	0.68	0.42	1.10	0.113	694	1.13	0.79	1.62	0.495
17	rs727428	T	*SHBG*	1440	1.03	0.99	1.06	0.079	740	0.81	0.62	1.06	0.127	700	1.05	0.83	1.34	0.665
17	rs1641549	T	*TP53*	1430	1.00	0.97	1.03	0.977	735	0.95	0.72	1.26	0.707	695	0.92	0.71	1.21	0.561

Note: OR—odds ratio; 95%CI–95% confidence interval. All results were obtained for the additive model after adjusting for covariates (*—no covariates were used). P_perm_ values < 0.025 are shown in bold.

**Table 3:** Haplotypes of *FSHB* gene SNPs and the risk of EnH_s_ among BMI < 25 and BMI ≥ 25 females.

SNP		Frequency		OR	*p*	P_perm_
rs11031002	rs11031005	Endometrial Hyperplasia	Controls			
female with BMI < 25						
A	C	0.066	0.114	0.64	0.057	-
T	C	0.011	0.015	0.44	0.196	-
A	T	**0.005**	**0.014**	**0.01**	**2 × 10^−6^**	**3 × 10^−5^**
T	T	**0.918**	**0.858**	**2.31**	**0.0004**	**0.003**
female with BMI ≥ 25						
A	C	0.067	0.112	0.71	0.106	-
T	C	**0.008**	**0.035**	**0.12**	**5 × 10^−5^**	**3 × 10^−4^**
A	T	**0.005**	**0.034**	**0.04**	**8 × 10^−7^**	**1 × 10^−6^**
T	T	**0.920**	**0.819**	**3.27**	**4 × 10^−9^**	**1 × 10^−6^**

Note: OR—odds ratio; *p*–significance level. The results were obtained by the logistic regression analysis with adjustment for covariates. Statistically significant results are highlighted in bold, taking into account the permutation test (1000 permutations were performed).

**Table 4:** SNP × SNP interactions significantly associated with endometrial hyperplasia among BMI < 25 and BMI ≥ 25 female.

N	SNP × SNP Interaction Models	NH	*beta*H	WH	NL	*beta*L	WL	P_perm_
**female with BMI < 25**								
Two-order interaction models (*p* < 6.81 × 10^−5^)								
1	rs11031002 *FSHB* × rs11031005 *FSHB*	1	0.84	11.54	2	−0.86	10.21	0.001
Three-order interaction models (*p* < 3.91 × 10^−5^)								
2	rs11031002 *FSHB* × rs117585797 *ANO2* × rs11031005 *FSHB*	1	0.79	11.85	1	−3.36	16.91	<0.001
Four-order interaction models (*p* < 1.50 × 10^−5^)								
3	rs11031002 *FSHB* × rs117585797 *ANO2* × rs11031005 *FSHB* × rs34670419 *ZKSCAN5*	1	0.71	11.43	1	−3.17	14.37	<0.001
**female with BMI ≥ 25**								
Two-order interaction models (*p* < 8.95 × 10^−6^)								
1	rs11031002 *FSHB* × rs11031005 *FSHB*	1	1.17	33.75	2	−2.40	37.02	<0.001
2	rs117145500 *CHD9* × rs11031002 *FSHB*	1	0.63	13.55	1	−1.13	19.72	<0.001
Three-order interaction models (*p* < 3.34 × 10^−9^)								
3	rs11031002 *FSHB* × rs112295236 *SLC22A10* × rs11031005 *FSHB*	1	0.92	27.39	2	−2.69	38.36	<0.001
4	rs11031002 *FSHB* × rs117585797 *ANO2* × rs11031005 *FSHB*	1	1.04	29.52	2	−2.54	37.75	<0.001
5	rs11031002 *FSHB* × rs11031005 *FSHB* × rs148982377 *ZNF789*	2	1.10	30.70	2	−2.63	36.22	<0.001
6	rs11031002 *FSHB* × rs11031005 *FSHB* × rs34670419 *ZKSCAN5*	1	0.87	23.06	2	−2.45	34.97	<0.001
Four-order interaction models (*p* < 5.02 × 10^−9^)								
7	rs11031002 *FSHB* × rs117585797 *ANO2* × rs112295236 *SLC22A10* × rs11031005 *FSHB*	1	0.86	25.67	2	−2.68	38.06	<0.001
8	rs11031002 *FSHB* × rs117585797 *ANO2* × rs11031005 *FSHB* × rs148982377 *ZNF789*	2	0.97	26.82	2	−2.80	36.49	<0.001
9	rs11031002 *FSHB* × rs112295236 *SLC22A10* × rs11031005 *FSHB* × rs148982377 *ZNF789*	2	0.87	25.02	2	−2.79	36.02	<0.001
10	rs11031002 *FSHB* × rs117585797 *ANO2* × rs11031005 *FSHB* × rs34670419 *ZKSCAN5*	1	0.83	22.19	2	−2.60	35.57	<0.001
11	rs11031002 *FSHB* × rs112295236 *SLC22A10* × rs11031005 *FSHB* × rs34670419 *ZKSCAN5*	2	0.92	27.96	2	−2.59	35.05	<0.001
12	rs11031002 *FSHB* × rs11031005 *FSHB* × rs148982377 *ZNF789* × rs34670419 *ZKSCAN5*	2	0.84	21.27	2	−2.56	34.18	<0.001

Note: NH—number of significant high risk genotypes in the interaction; *beta*H—regression coefficient for high risk exposition in the step2 analysis; WH–Wald statistic for high risk category; NL—number of significant low risk genotypes in the interaction; *beta*L—regression coefficient for low risk exposition in the step2 analysis; WL–Wald statistic for low risk category; P_adj-perm_—permutation *p*-value for the interaction model (1.000 permutations). The results were obtained using the MB-MDR method with adjustment for covariates.

**Table 5:** Presumptive functional significance of the EnH_s_-associated SNP rs148982377 [T>C] *ZNF789* (and proxy rs34670419 [G>T] *ZKSCAN5*) in women with BMI ≥ 25 in adipose tissue.

SNP	Mesenchymal Stem Cell-Derived Adipocyte Cultured Cells	Adipose-Derived Mesenchymal Stem Cell Cultured Cells	Adipose Nuclei
rs148982377 [T>C] *ZNF789*		H3K9ac_Pro	H3K4me1_Enh H3K9ac_Pro
rs34670419 [G>T] *ZKSCAN5*	H3K4me3_Pro	H3K4me3_Pro	H3K4me3_Pro

Note: indicated by their location in the regions of histone proteins marking enhancers (H3K4me1), promoters (H3K4me3) and active promoters (H3K9ac) according to HaploReg v.4.2.

## Data Availability

The data generated in the present study are available from the corresponding author upon reasonable request.

## Supplementary Materials

The following supporting information can be downloaded at: https://www.mdpi.com/article/10.3390/life16060937/s1, Table S1: The GWAS data about associations of the studied candidate genes polymorphisms with the level of sex hormones; Table S2: The regulatory potential of the studied SNPs; Table S3: The allele and genotype frequencies of the studied SNPs in the endometrial hyperplasia and control groups with BMI < 25; Table S4: The allele and genotype frequencies of the studied SNPs in the endometrial hyperplasia and control groups with BMI ≥ 25; Table S5: Genotype combinations associated with endometrial hyperplasia among BMI < 25 female; Table S6: Genotype combinations associated with endometrial hyperplasia among BMI ≥ 25 female; Table S7: Regulatory effects of the EH-associated loci and SNPs in high LD (r^2^ ≥ 0.80); Table S8: The eQTL effects of the EH-associated SNPs in various tissues/organs; Table S9: eQTL values of SNPs in high LD (r^2^ ≥ 0.80) with the EH-associated polymorphisms; Table S10: The sQTL effects of the EH-associated SNPs in various tissues/organs; Table S11: sQTL values of SNPs in high LD (r^2^ ≥ 0.80) with the EH-associated polymorphisms.

## Abbreviations

The following abbreviations are used in this manuscript:

BMI	Body mass index
EH_s_	Simple endometrial hyperplasia without atypia
SNP	Single nucleotide polymorphism
GWAS	Genome-wide studies
OR	Odds ratio
SHBG	Sex hormone-binding globulin
LH	Luteinizing hormone
FSH	Follicle-stimulating hormone
LD	Linkage disequilibrium
